# Within-subject and between-subject variability in responses to a mind-body intervention in electrophysiological brain activity

**DOI:** 10.3389/fpsyg.2026.1872803

**Published:** 2026-07-22

**Authors:** Nina Zech, Martin Busch, Veronika Jaeger, Ernil Hansen

**Affiliations:** 1 Department of Anesthesiology, University Hospital Regensburg, Regensburg, Germany; 2 Private Practice, Dunningen-Lackendorf, Germany

**Keywords:** bispectral index, chronotype, hypnotic susceptibility, mind body intervention, trance

## Abstract

**Introduction:**

Mind-body interventions (MBIs) increasingly used in health care include alterations in the ordinary state of consciousness that might be essential for their effects on psychological and physical patterns encoded in the unconscious. Little is known about the effects of repeated practice or the time of the repetitions.

**Methods:**

EEG-derived Bispectral-Index (BIS) is clinically used to monitor anesthetic depth, a deviation from the normal waking consciousness. We used BIS-monitoring in six participants who repeated a MBI ten times within 3–4 days at different times of day.

**Results:**

BIS values decreased significantly from an awake baseline of 97.5 to a mean of 83.2, showing a magnitude of reduction similar to that observed during the standardized hypnosis induction of a suggestibility test. Marked drops in BIS occurred during the breaks before and after the four tasks of the MBI and within certain tasks. There was a steady decline in BIS during the 30-min MBI, and some deepening with increasing exercise. Changes in BIS were significantly more pronounced when the MBI was performed at midday compared to mornings, particularly in individuals with an eveningness chronotype.

**Discussion:**

The reproducibly observed decrease of BIS during the MBI we take as surrogate for involved trance states in support of the hypothesis that this facilitates access to the unconscious and modulation of physiological and psychological programs encoded there. The within-subject variability in the corresponding EEG-derived BIS with repetitions of the MBI is unexpectedly high, although still lower than between-subject variability. Some improvement was observed with repeated practice, although not in a linear relationship. The dependence of these effects on time of day warrants further investigation.

## Introduction

Most psychotherapeutic and mind-body interventions (MBI) rely on repetition, as they are regarded as learning processes (Erickson and Rossi, 1980; Yan et al., 2025). Mind-body instructions are often given via recordings and regular repetition of the exercises is recommended for patients. Similarly, in hypnotherapy patients are usually trained in self-hypnosis to continue successful therapeutic approaches. Both therapists and participants expect similar effects with repeated practice. In particular, the accompanying trance state is thought to recur at a comparable extent and quality. Most studies examining the effects of such interventions involve groups of participants. Between-subject variability is observed, and the distribution of effects is calculated in order to draw conclusions about an average population. Less information is available about the variability of a specific effect in an individual subject with repetition of the intervention, except that some learning effect is assumed. Moreover, time-of-day dependency is rarely considered or studied in relation to effects and effectiveness of both mind-body-interventions and hypnotherapy, despite demonstration of physiological and psychological circadian or ultradian rhythms (Ayyar and Sukumaran, 2021; Kleitman, 1982; Lightman and Conway-Campbell, 2024; Rossi, 1982). Postprandial activation of the parasympathetic nerve system and a shift of physiological activity toward digestion may also have an impact on attention, suggestibility, and efficacy of interventions (Diekmann et al., 2019).

We recently published a study on trance states during a mind-body intervention based on measurements of the bispectral index (BIS) with an anesthesia depth monitor (Zech et al., 2026). In addition to the measurements on a study population of 54 volunteers reported therein, we applied the same MBI to 6 participants with 10 repetitions of the measurement. In these within-subject analyses the effects of repetition and time of day were investigated. Given the lack of prior repeated-measures electrophysiological datasets in this field, the present study should be considered exploratory.

Usually, research on MBIs or hypnotherapy focuses on their effects. However, the goals and objectives pursued by these interventions are very diverse, varied, and often complex such as pain reduction. Noteworthy, deviation from waking consciousness, in focus here, is a more general parameter and important when considered essential for reaching the unconscious mind to modulate physiological and psychological patterns encoded there (Landry et al., 2014; Peter, 2024).

## Materials and methods

### Study design

For this prospective observational exploratory study, participants were recruited between November 2024 and January 2025. Exclusion criteria were as follows: age < 18 or > 77, insufficient language proficiency, significant hearing impairment, inability to lie still and comfortably on the floor, as well as major pre-existing medical conditions such as epilepsy, neurological or psychiatric disorders, or current use of psychotropic medication. The study was approved by the local ethics committee of the University of Regensburg as an amendment to the votum 13-101-0040. Participants were enrolled after providing written informed consent.

To investigate repetition effects, 10 repeated measurements of the same intervention were considered feasible and compatible within a time frame of 3–4 days. Due to the lack of prior repeated-measures electrophysiological datasets in hypnosis- or trance-related interventions, no reliable basis for a formal power analysis specific to the present study design was available. The present exploratory study therefore focused on repeated within-subject measurements, resulting in a total of 58 BIS recordings. This approach was oriented toward the design of a previous study 54 participants with one measurement each, according to a power analysis (Zech et al., 2026)).

### Mind-body intervention

The mind-body intervention (MBI) tested was developed according to Martin Busch (Zech et al., 2026). The 30-min intervention recorded was specifically recorded for this and the previous study. It consists of an introduction followed by a reflection phase (break 0) and instructions for four tasks with subsequent rest periods (Table 1). The text of the instructional audio file is provided in Supplementary material.

**TABLE 1:** Phases of the mind-body intervention and their duration.

Time (s)	Duration (s)	Intervention	Explanation
000	235	Introduction	
235	95	Break 0	Body scan, reflections
330	280	Task 1	Elbow/Foot
610	95	Break 1	
705	239	Task 2	Eyes/Head
944	112	Break 2	
1,056	308	Task 3	Breathing
1,364	76	Break 3	
1,440	260	Task 4	Eyes diagonal
1,700	86	Break 4	
1,786	24	Exit	Eyes open, reorientation
1,810		End	

Based on the Moshe Feldenkrais’ approach that includes Awareness Through Movement and Functional Integration to develop new movement patterns (Buchanan and Ulrich, 2001; Martin et al., 2024), the Martin Busch approach encourages experiments with variations of a movement instead of repeated practice of complex movement patterns. Directing attention through a movement suggestion that triggers different possible actions leads to the exploration of different variations (see the “Introduction” in the text given in the supplement). To achieve this, language is precise in the description of “What” is done, and open to individual interpretation of “How” it is done. The instructions used here with the present audio file includes pressing one foot and the opposite elbow against the floor (Task 1), movement of the eyes separately or together with the head from side to side (Task 2), breathing with the hands uncrossed or crossed on upper or lower ribs (Task 3), and eye movements together with the imagination of a diagonal between shoulder and opposite hip (Task 4). The letter task includes the inexecutable suggestion of eyes moving forwards and backwards separately in the head.

Movements are performed in rhythm with the breath, since breathing is also a movement of diaphragm and ribs, and controlled both consciously and unconsciously, therefore it serves as a bridge to the unconscious. Moreover, this clock generator allows for individual and condition-adjusted pacing of the movements, in contrast to exercises, where breathing follows the movements. Even whether a movement goes with breathing out vs. breathing in makes a difference. The participant is encouraged to observe “how you do what you do,” and again after variations of the movements. Thus, the central nervous system receives novel information, and the subsequent breaks allow the brain to compare the new information with its previous experiences, integrate new information and confirm what it already knows to optimize processes instead of practicing complex patterns. The psychological and neurophysiological mechanisms underlying the MBI is that elements derived from hypnosis (inward attentional focus, mindful body awareness, breathing-induced relaxation, mental overloading, confusion, sensitivity to minimal cues, involuntariness and acceptance of loss of voluntary control) induce a trance state that allows for access to the unconscious, where programs of movement, physiological and psychological reactions are encoded in patterns.

For calculations, the phases of the MBI were divided into time blocks of approximately equal duration (1.5–2 min). The phase corresponding to the deepest BIS value (“deepest phase”) was determined for each participant and across all participants. The starting time of each MBI repetition was chosen to obtain measurements in the morning (6–11 a.m.), at midday (11 a.m–4 p.m.), and in the evening (4–10 p.m.), as well as pre- and postprandial.

### BIS monitoring

During each session of the MBI, an EEG-derived index was continuously recorded by a anesthesia depth monitor, specifically, the Bispectral Index Scale monitor (BIS-monitor, VISTA^®^ monitoring system; Anandic Medical Systems, Switzerland). The BIS index is a numerically processed, clinically validated EEG parameter (Punjasawadwong et al., 2014). Unlike traditional processed EEG parameters derived from spectral analysis, the BIS index is derived utilizing a composite of multiple advanced EEG signal processing techniques, including bispectral analysis, power spectral analysis, and time domain analysis. The key EEG features extracted from the database analysis include the degree of beta or high frequency (14–30 Hz) activation, the amount of low-frequency synchronization, the presence of nearly suppressed periods within the EEG, and the presence of isoelectric periods within the EEG (Sigl and Chamoun, 1994). The exact algorithm for weighting and mathematical combination of these components to a single BIS value is unknown, as it has not been publicly disclosed by the manufacturer for patent protection. Nevertheless, despite these limitations BIS monitoring is widely used in clinical studies and practice. The BIS sensor is a single-use component consisting of an adhesive pad with fixed electrodes. The sensor is placed with the central electrode at the center of the forehead, half a centimeter above the bridge of the nose, two electrodes above the left eyebrow, and an electrode midline between the edge of the eye and the hairline.

Baseline measurements of “BIS awake” were recorded for several minutes before and after the MBI. For analysis the first 90 s of the BIS-recording were discarded to account for system latency. The measurement was conducted in a quiet room to avoid any disturbance, and under controlled lighting and temperature conditions. For the MBI, participants first sat on the floor on a comfortable mat with a pillow. After the introduction via ear-phones the participant lay down on the mat to follow the instruction of the audio file (for the test setting see Zech et al., 2026). Measurement times were selected to obtain data for “morning,” “midday,” “evening,” “preprandial,” and “postprandial,” respectively, in each of the test subjects twice. “Effect of BIS” was calculated as the difference between BIS awake and BIS values during the MBI session. Relative SD was calculated according to the formula: rSD = SD/mean effect of BIS (%).

For comparison of BIS-changes to a formal hypnosis induction, initially a suggestibility test under BIS-monitoring was performed by each of the participants, namely the HGSHS-5 (Riegel et al., 2021; Zech et al., 2024). This shortened version of the Harvard Group Scale of Hypnotic Susceptibility (Shor and Orne, 1963) includes a standardized trance induction commonly used worldwide and allows classification in “low”- and “high”-suggestibles. In addition to these suggestibility groups, participants were classified into “Larks” ( > 59 points) and “Owls” ( < 41 points) based on the morningness-eveningness questionnaire (Horne and Ostberg, 1976).

### Statistical analyses

Variables were tested for normal distribution using the Kolmogorov-Smirnov-Lilliefors test and by visual inspection of histograms. Accordingly, we present normally distributed variables with mean ± standard deviation (SD) and use a paired or unpaired Student’s *t*-test, as appropriate. Non-normally distributed data are presented as median and interquartile range (IQR) and compared using the Mann–Whitney *U*-test.

To analyze within-subject variability of BIS values and compare it with the between-subject variability reported in the previous study (Zech et al., 2026), means were calculated for every second of the intervention or an intervention phase across the 10 MBI repetitions. SD was treated exactly the same way to show variability in the repetitions, not during the measurement of one MBI application. Only then a mean for the six test persons was calculated. Since SD varies with the extent of an effect (a low effect shows small SD), a “relative SD” (rSD) was calculated using the formula: rSD = SD/effect size, namely rSD = SD/Effect on BIS = SD/(BIS awake - BIS during intervention).

To account for the repeated-measures structure of the data, a linear mixed-effects model (LMM) was conducted with “MB deepest phase” as the dependent variable. Fixed effects included time of day (morning, midday, evening), chronotype (morning type vs. evening type), and their interaction. Participant was included as a random intercept to account for within-subject dependency due to repeated measurements across the ten intervention sessions. Model parameters were estimated using restricted maximum likelihood (REML). A significance level of *p* < 0.05 was considered statistically significant for all tests. All analyses were conducted using IBM SPSS Statistics, Version 31.

## Results

### Baseline characteristics

The participants consisted in four women and two men, ranging in age between 27 and 76 years. Two participants were classified as high and four as low suggestible in the suggestibility test. Three participants were classified as “larks” and three as “owls” based on the Morningness–Eveningness Questionnaire (Horne and Ostberg, 1976). Of the six participants, ten measurements were analyzed for five individuals, whereas only eight measurements were available for one individual because of technical problems during BIS registration. Of the total 58 measurements, 14 were conducted in the morning, 25 at midday, and 18 in the evening. In addition, the temporal relation to meals was defined as preprandial for 13 measurements and postprandial for 15 measurements.

### Individual BIS readings

**FIGURE 1:**
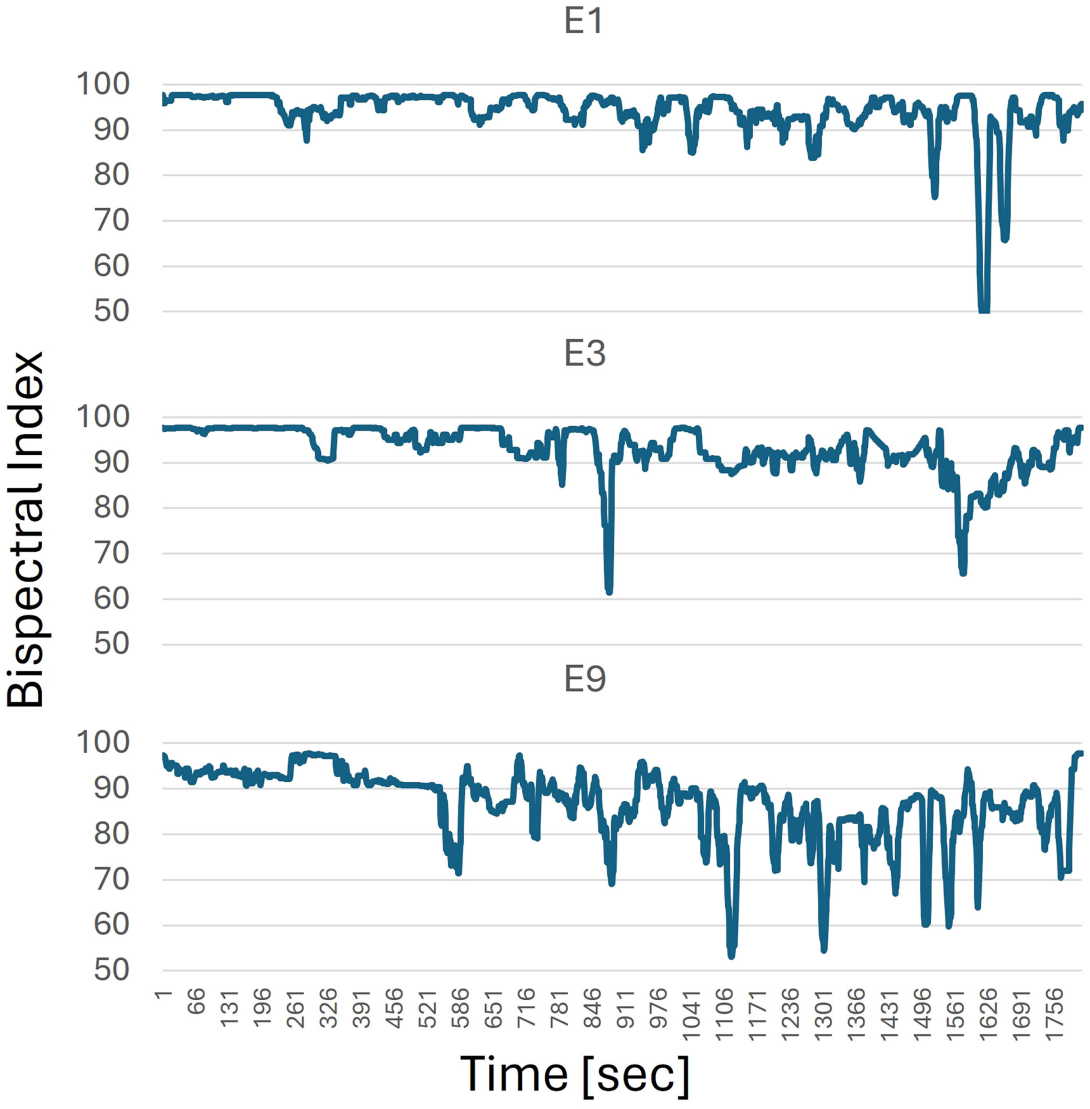
Individual BIS readings during MBI. Bispectral Index was monitored during repeated MBI. Three examples out of 10 repetitions are given for one participant (E).

Significant deviation from BIS awake was observed in all measurements. In individual cases BIS decreased to around 40. The individual BIS recordings during repetition of the same MBI in each tested person revealed a high variability. Three examples of one participant are given in Figure 1. While in the first applications in each of the subjects BIS values close to the individual “BIS awake”- value was observed during the “introduction” given at the beginning of the audio file, in later repetitions decreases in BIS occurred already at this part of the intervention. For an example, compare the first 4 min in E1 and E9 in Figure 1. Moreover, greater fluctuations in BIS were observed with further repetitions of the MBI.

### Individual participants

**FIGURE 2:**
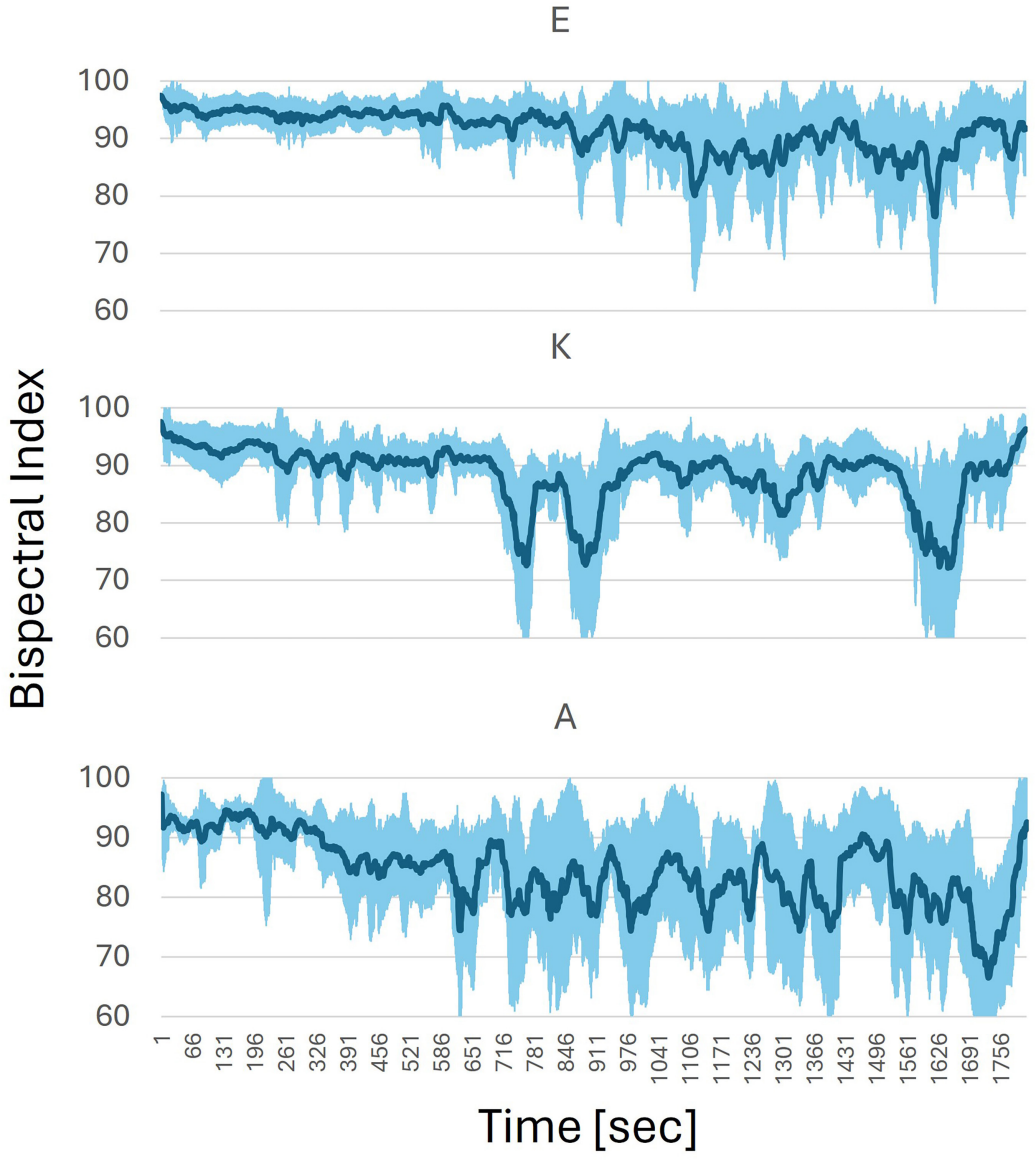
Cumulative BIS readings during MBI of three participants. Bispectral Index was monitored during repeated MBI. Cumulative curves were calculated from 10 repetitions for every participant. Three examples out of six participants are given.

The composite graph of all 10 measurements of participant E is shown in Figure 2, together with those of two other subjects. The individual results of the 6 tested persons differed markedly. However, similarities were also observed, such as the steady decline in BIS during the MBI, and in BIS drops at certain MBI phases.

### BIS-curve during MBI and its variability

**FIGURE 3:**
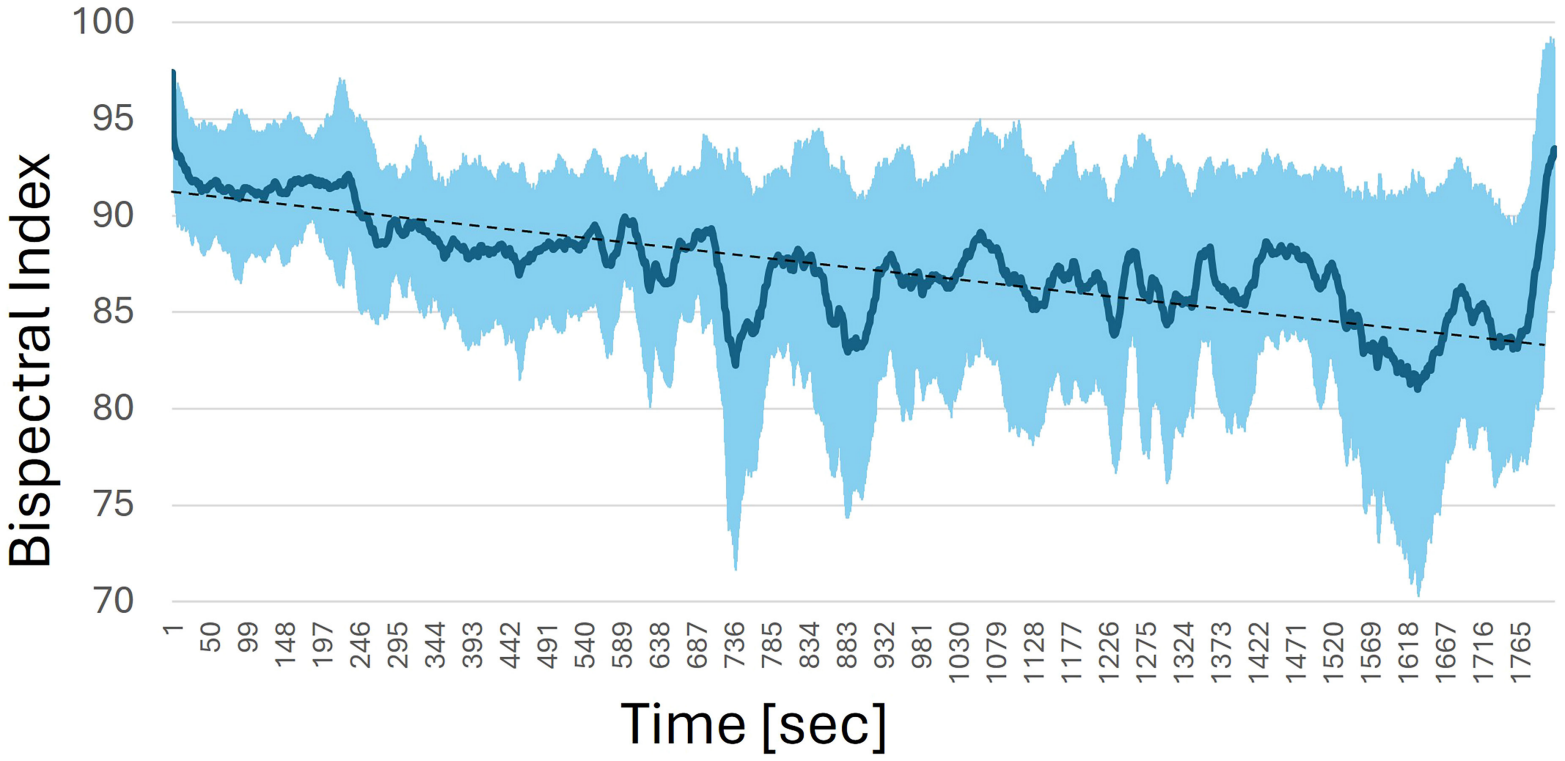
Cumulative BIS readings during MBI of all six participants. Bispectral Index was monitored during repeated MBI. Cumulative curves were calculated from 10 repetitions for every participant. Means and SDs of all 6 participants are shown. Dotted line: trend line.

**FIGURE 4:**
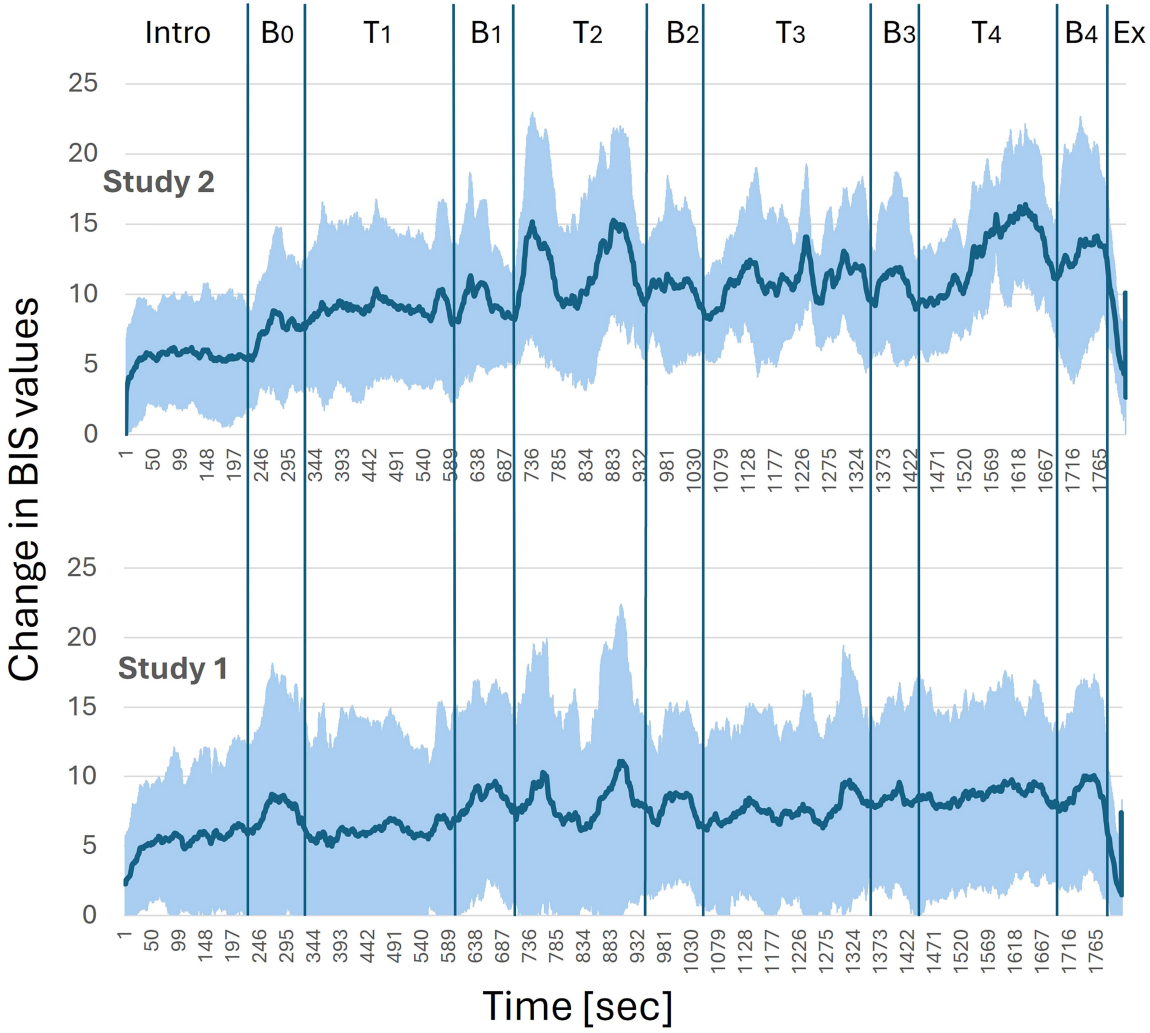
Change in BIS values during MBI of all six participants. Comparison of the present study on measurement repetitions (study 2) with a previous study on single measurements of 54 participants (study 1) (Zech et al., 2026).

The composite graph of all 6 participants is shown in Figure 3. The trend line indicates a gradual decrease in BIS values during the MBI. An alternative, reciprocal representation is given in Figure 4 where the effect of the MBI on BIS is represented by the difference between the respective BIS value and the baseline (BIS awake). Compared to the preceding study involving 54 participants with a single MBI application (see Figure 4) the range of dispersion (SD) was smaller in the present study with repeated MBI sessions (Figure 4, upper part). Visually, the dispersion of BIS values appeared lower in the present repeated-measures dataset than in the previous between-subject dataset, although the substantially different study designs preclude direct inferential comparison. The different phases in the course of the MBI are also marked in this graph, in particular the four tasks and the five breaks. Significant changes in BIS were observed, particularly during each break. Overall, the MBI phase with deepest BIS level, or the highest effect on BIS, respectively, was identified at the second half of task 4 (T4b). In 27 of 58 (47%) evaluable measurements T4b was the “deepest phase.” Moreover, this time course of BIS in 10 repetitions showed a higher effect than in the previous study (compare study 1 and study 2 in Figure 4).

The extent of the effect of the MBI on BIS and its variability is also shown in Table 2 for the complete intervention and the phase with the deepest values of BIS, namely T4b. On average, the BIS values were lower in the current study on exercise effects. At the same time, the variability in the effect on BIS was lower, as indicated by the SD. It should be noted that the SD in Table 2 is not the SD of means, usually presented in “mean ± SD,” but the mean of SDs, i.e., the variation at every second during the 10 MBI repetitions. Moreover, to correct for the fact that variability increases with greater magnitude of an effect, rSD is provided, the SD relative to the BIS reaction. This further characterization of the magnitude of this variability (Table 2). While in the preceding study variation amounted to 95% of the effect value, in the present study the value variability was only 52% of the BIS change across the entire time of the MBI.

**TABLE 2:** Effects of MBI or HGSHS-testing on BIS and its intra- and between-subject spread width.

	Mean in complete MBI (1,810 s)			Mean in MBI task 4b (132 s)			Mean in HGSHS task 1a (133 s)		
	BIS	SD	rSD	BIS	SD	rSD)	BIS	SD	rSD
10 repet. 6 prs.	87.35	5.08	52%	83.19	7.58	55%	84,69	8.01	62%
54 prs. (Zech et al., 2026)	90.09	6.88	95%	88.50	6.61	87%	85.15	7.22	75%

The comparison between the present repeated-measures dataset and the previous between-subject dataset is intended for descriptive purposes only, given the substantial differences in study design. For analysis of intra- and between-subject variability the mean for every second of the intervention or intervention phase, respectively, of the 10 repetitions was calculated. In the same way, the SDs for every second were calculated. Only then the means of the mean values and the SDs of six test subjects were derived. Note that therefore the SD given in the table does not represent the variation of values during each measurement, but that of the repeated measurements at each second of the MBI repetitions. Similarly, the SDs in the preceding study (line 2) represent the variation of the 54 participants at any second of the intervention, not that during each measurement. Since the variability of BIS values increases with increase in the deviation from the baseline BIS wake, the relative SD is given in addition according to the formula: rSD = SD/effect size, namely rSD = SD/(BIS awake - BIS during intervention).

In addition, Table 2 shows a comparison between the effect of the tested MBI on BIS with the effect of a standardized hypnotic induction as part of HGSHS, the test for suggestibility. The phase with the deepest BIS values during the MBI, namely T4b, and during HGSHS testing (T1a) resulted in about the same BIS levels.

### Repetition-related effects

**FIGURE 5:**
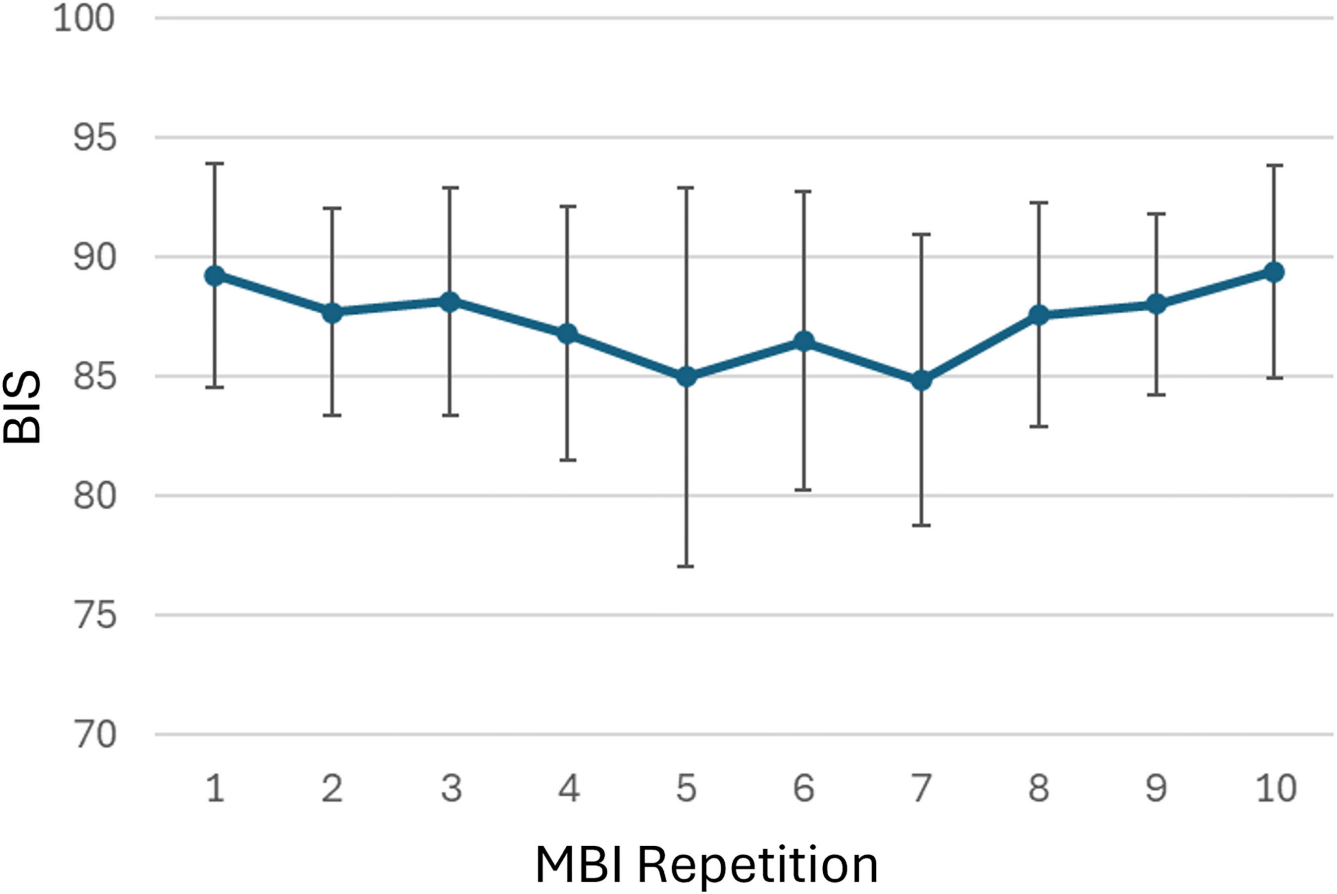
Practice effect with MBI repetitions. BIS during all breaks (7.7 min period) of MBI is shown (*n* = 6, mean SD) for the 10 repetitions of the MBI.

An increased reaction, i.e., lower BIS values, was seen with repetitions of the MBI in several parameters, such as BIS during total MBI, common deepest phase (T4b), or individual deepest phase. However, linear regression analyses did not reveal a significant relationship. For maximum significance, the parameter “all breaks” is used in Figure 5, encompassing a longer time period. A trend toward lower BIS values, i.e., a higher effect on BIS, was observed for the first 5–7 repetitions, followed by a decrease of this improvement.

### Effect of time of day and participants’ chronotype

**FIGURE 6:**
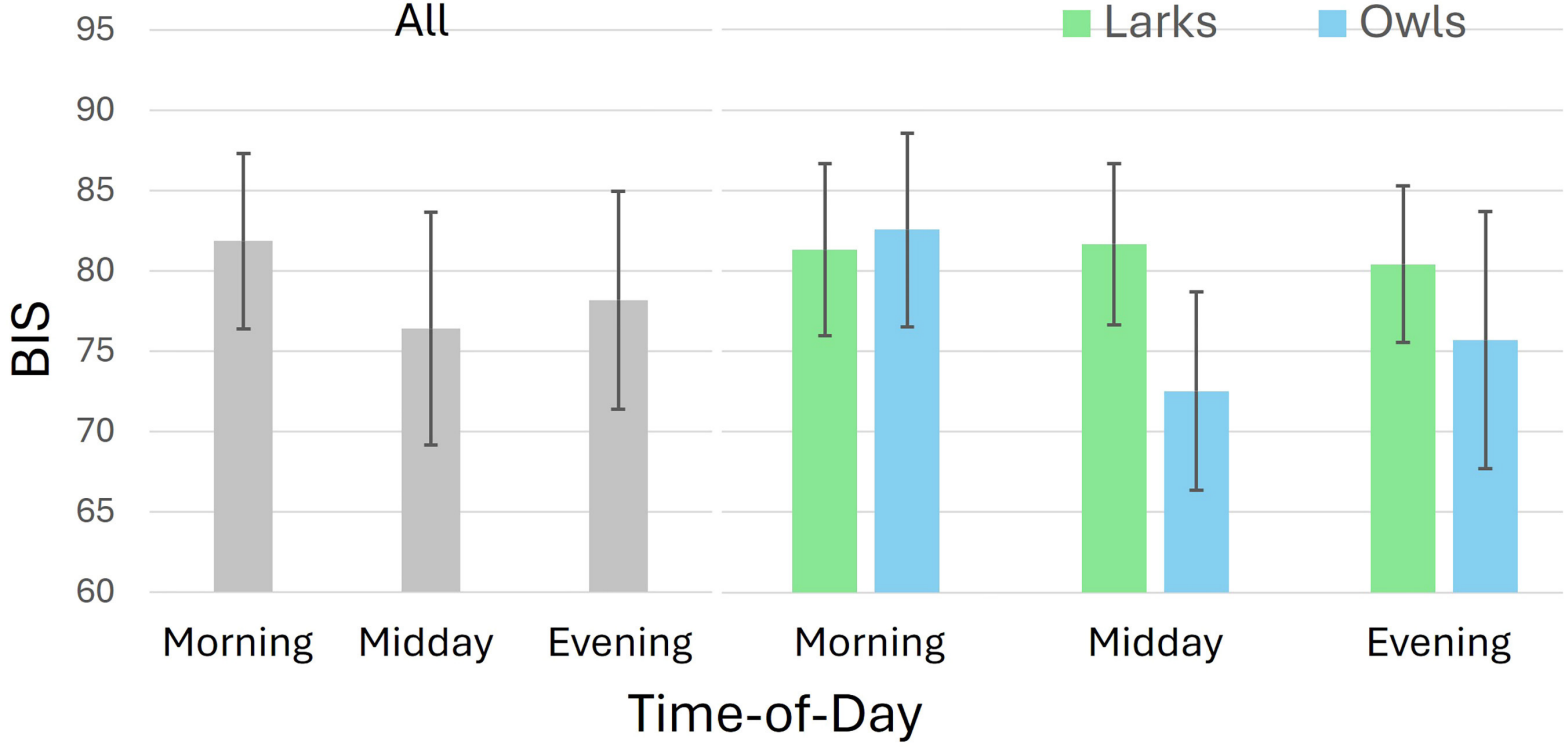
Exploratory associations between time of day, chronotype, and BIS response. BIS during individual deepest phase (ca. 2 min period) of MBI is shown (mean ± SD) Morning = 6–11 a.m. (*n* = 15), midday = 11 a.m–4 p.m. (*n* = 25), evening = 4 p.m–10 p.m. (*n* = 18). Larks: > 59 pts., Owls: < 41 pts. in morningness-eveningness questionnaire (Horne and Ostberg, 1976).

The consecutive MBI repetitions were categorized by the time-of day, and study participants were grouped according to their chronotype into morningness and eveningness type. The results with regard to the deepest BIS values are shown as grey columns in Figure 6. The linear mixed-effects analysis revealed a significant effect of time of day on MB deepest phase. The consecutive MBI repetitions were also indexed by the time-of day, namely grouped to morning, midday and evening. Compared with the evening reference condition, measurements obtained at midday were significantly lower [*b* = −6.91, SE = 2.21, *z* = −3.13, *p* = 0.002, 95% CI (−11.23, −2.58)]. In contrast, morning measurements did not significantly differ from evening measurements (*b* = 3.08, *p* = 0.245). The main effect of chronotype was not significant (*b* = 0.88, SE = 4.51, *z* = 0.19, *p* = 0.846), indicating that morning- and evening-type participants did not differ overall in BIS values. However, a significant interaction between time of day and chronotype emerged for midday assessments [*b* = 8.27, SE = 3.10, *z* = 2.67, *p* = 0.008, 95% CI (2.19, 14.35)]. This finding suggests that the effect of midday assessment differed between morning- and evening-type participants. No significant interaction was found for morning assessments (*p* = 0.494).

### Pre/postprandial effect of MBI on BIS

Of the analyzed parameters (complete MBI, all breaks, task 4b, deepest phase) only task 4b showed a trend (*p* = 0.160) toward a difference of BIS reaction to MBI pre- (median 84.6, IQR 8.4) vs. postprandial (median 81.8, IQR 9.1).

## Discussion

### Individual reaction of BIS to MBI

The results of the present study are broadly consistent with findings from our previous study reporting EEG-related changes during the same MBI (Zech et al., 2026). The variability in the time course of BIS and the extend of BIS-decline for different individuals observed there was expected. However, the high within-subject variability in the present study, when the MBI was repeated by the same person, was rather unexpected. Exercise and practice of an intervention are fundamental in the concepts of MBI or psychotherapeutic interventions like hypnosis (Erickson and Rossi, 1980; Yan et al., 2025). An individual’s reaction to the exercise instructions is regarded as a fixed factor and characteristic of that person. Different persons might react differently, but a specific person with his individual predisposition, education, and history is considered to show a fairly consistent reaction to a given instruction and performance of the intervention. In contrast, extensive variability in an individual‘s response to the MBI was observed in the present study (see examples in Figure 1). This variability may reflect a combination of factors, including physiological fluctuations, differences in attentional engagement, habituation effects, fatigue, or other intervention-related processes. Despite the enormous fluctuation in responses, the within-subject variability still was smaller than those between-subjects. Obviously, factors other than individual predispositions play a role, perhaps the time of day or acute events and challenges., Although technical measurement instability cannot be completely excluded, BIS recordings were obtained under standardized recording conditions using clinically established monitoring equipment.

It is noteworthy that during the MBI in many cases BIS reached levels down to around 40, well in the region that is considered optimal and is the targeted depth of anesthesia for surgery BIS 40–60 (Heavner et al., 2022). However, this does not imply comparable phenomenological or physiological states. Phases of MBI with deep BIS levels were the breaks before and after the tasks, where self-reflection and body scan are suggested, basic components of both mindfulness exercises and hypnotherapy (Gan et al., 2022; Peter, 2024). The common deepest phase was T4b, an impossible task with the request for forward-backward movements of the eyes representing a strong trance induction by confusion (Erickson, 1964). Interestingly, its BIS-deepening effect was more pronounced in this study focusing on within-subject effects than during the previous study (Zech et al., 2026).

### Repetition-related changes in BIS values

There are tentative indications of a learning effect both with time during each intervention described by the steady decline in BIS (see trend line in Figure 3), and by the deeper BIS levels with repetition of the intervention (see Figure 5), respectively. The former might be seen as a deepening of trance with time (Mann and Sanders, 1995), or a learning effect during the four tasks of the MBI and the inserted breaks. Repeated applications of the MBI were associated with slightly lower BIS values. However, no linear relationship over the 10 repetitions could be found, rather a learning effect with the first few repetitions that vanished with further exercises. Statistically, only a trend to deeper BIS levels during the first 5–7 repetitions was observed, and primarily for the parameter “all breaks,” which includes more data (covering 7.7 min) than the common or individual deepest MBI phase (of about 2 min). Some participants felt bored with the late repetitions or distracted to other mental activities. Another reason could be a shift of attention to different parts and phases of the MBI with ongoing exercise, or the confounding impact of other factors of motivation and conditions, like time of day of the intervention. Further factors like sleep quality, caffeine intake, fatigue, prior activity, or meal timing may have confounded the results.

### Time -of-day dependency

Chronobiological rhythms play a pivotal role in cellular, physiological and psychological processes (Lightman and Conway-Campbell, 2024; Ulfah Suhardita et al., 2025). Their significant impact on physical activities such as sports, on physiological functions such as metabolism or immune competence, and on mental functions such as cognition or memory has been recognized, as well as the consequences of their disruption for the development of diseases (Foster and Kreitzman, 2014). Besides common circadian cycles of sleep-wake, hormone levels, e.g., of cortisone, body temperature or metabolism, differences between different people as a trait have been observed. For instance, “morningness” and “eveningness” chronotypes can be distinguished (Montaruli et al., 2021). The results of the present study suggest that the BIS during the deepest phase of the MBI is influenced by the interaction between circadian preference and assessment timing rather than by chronotype alone. Specifically, midday assessments were associated with lower BIS values overall, but this effect differed significantly between morning- and evening-type participants. Different responses have been described for the chronotypes “larks” and “owls” with regard to hypnotic susceptibility or hypnotic depth. In that respect, our results are in line with a study that showed several points higher HGSHS scores for owls in the morning (8–10 a.m.) and for larks in the late afternoon (4–6 p.m.) (Lippincott, 1992). Furthermore, with a short hypnotic induction higher hypnotic depth was achieved for larks in the morning and for owls in the late afternoon (Mann and Sanders, 1995). These seemingly contradictory findings could be explained by interference of the activity state with a trance-promoting rest state. With regard to biological rhythms in human consciousness and performance it should be noted that in our study an objective measure was used, namely changes in EEG, instead of subjective, mostly self-reported variables. Our results support the assumption that chronobiological factors may modulate responses to mind–body interventions depending on the timing of assessment or practice. Given the relatively small sample size, these findings should be interpreted cautiously. Nevertheless, the use of a linear mixed-effects approach appropriately accounted for repeated measurements and individual variability, providing preliminary evidence for time-dependent chronobiological influences in the context of repeated mind–body interventions. Much more research is needed before the significantly deeper BIS level at midday observed in our study for all and specifically in night persons can have impact for hypnotherapy or MBI.

Besides circadian also ultradian rhythms with more than one cycle per day play a vital role in regulating physiological and psychological functions (Lightman and Conway-Campbell, 2024; Philippu, 2019). Starting from the observation of recurring phases of sleep with or without rapid eye movements (REM and non-REM sleep), N. Kleitman proposed a Basic Rest-Activity Cycle (BRAC) that also includes daytime (Kleitman, 1982). Moreover, E. Rossi described the regular sequence of periods of maximal responsiveness to a hypnotic induction and deepest trance states (Rossi, 1982). According to him, the “naturalistic approach” of M.H. Erickson to hypnosis based on a very accurate and sensitive observation of the patient and his state to utilize the natural periods of quietness and receptivity for trance induction and important suggestions, not on specific techniques or interventions. This biological rhythm consisting of 90–100 min of common everyday consciousness and about 20 min with low levels of alertness and vigilance and high production of mental imagery, the “common everyday trance,” is accompanied by fluctuations in EEG activity (Hayashi et al., 1994; Philippu, 2019), possibly by changes in brain lateralization (Shannahoff-Khalsa, 1991), and is flexible in timing and adaptable by situational demands and environmental conditions like stress. Because of the multiplicity of relevant phases during the day and because of the individual position and phase in the cycle of every test subject at certain times, the impact of this ultradian rhythm was not within the scope of the present study. However, it could well have impacted the results by contributing to the variability of measured effects.

### Limitations

The ideal comparison of between-subject and within-subject variability of effects would involve comparing a large number of participants undergoing a single MBI session with an equally large number of repeated measurements within a single individual. However, in the context of MBI or other psychological interventions the latter is impossible, at least within a reasonable time excluding other influencing factors. Therefore, in the present study the number of repetitions was limited to 10. This argument is supported by non-constant and non-linear effects of the repetitions (see Figure 5). Consequently, the results of this study contain both within-subject (10 repetitions) and between-subject (6 test subjects) variability of effects. The small number of participants limits generalizability and increases the risk of unstable subgroup estimates. Nevertheless, the repeated-measures design still allows preliminary observations regarding variability and repetition-related effects during the MBI.

The BIS value is a composite parameter of various EEG features, and the algorithm for combining and weighting of these variables is not disclosed by the monitor manufacturer (Heavner et al., 2022; Sigl and Chamoun, 1994). This represents an important methodological limitation Nevertheless, BIS monitoring has been widely used in clinical and research settings, and alterations in EEG have been described both for hypnosis and MBI (Duda et al., 2024; Lucas et al., 2025; Wolf et al., 2022). At the same time, hypnotic or other trance states are not characterized by changes in a single specific EEG wave length, specific EEG frequency band or a specific mixture of features of electrical brain activity (De Pascalis, 2024; Miltner et al., 2024). There is no unique fingerprint because of the complexity and due to the heterogeneity of processes involved in MBI or hypnosis (Rinaldi et al., 2025). Accordingly, the aim of the presented study was not to define a specific neurophysiological signature of MBI by EEG parameters and wave ranges, nor to compare MBI with other interventions involving trance states. Rather, BIS changes during the MBI and during the standardized hypnotic induction as part of the HGSHS suggestibility testing (Zech et al., 2023; Zech et al., 2026) were interpreted cautiously as indirect indicator of non-ordinary states of awareness and consciousness. The comparable BIS reduction during both interventions may rather suggests intervention-induced changes in consciousness to a similar extent. That fits with other similarities of MBI and hypnosis, potentially with relaxation as a mutual component of states of non-ordinary consciousness (Kokoszka et al., 1992).

Instead of defining specific states of consciousness, BIS actually is able to reproducibly and reliably, as demonstrated daily in clinical anesthesia routine, detect a deviation from normal wakefulness and restriction of the critical mind. The results of the present study support the assumption that such alterations can occur during MBI.

## Meaning and conclusion

This finding might be relevant for the hypothesis that communication with, and involvement of, the unconscious mind is crucial for facilitating favorable alterations in physiological and psychological patterns. Because those latter processes are anchored in the unconscious and hardly reached by the critical conscious mind or by deliberate decision-making (Hansen, 2024; Landry et al., 2014; Peter, 2024). Especially in hypnotherapy, surpassing resistances and the critical mind, accepting involuntariness, allowing changes to happen without intention are essential features. We propose that similar alterations of the conscious mind occur during mind-body of hypnotic interventions and are necessary for affecting the unconscious mind and processes programmed there.

Not only the therapeutically intended specific effects might change with repetition of the intervention, but also the access to the subconscious as suggested here. Similarly, effects of time of day are emerging, and should deserve further research and consideration. The observed BIS changes may contribute to a better understanding of altered attentional and awareness-related processes during MBIs and hypnosis-related interventions. Further studies using larger samples and more comprehensive neurophysiological approaches are needed to clarify the underlying mechanisms and their potential relevance for therapeutic effects.

## Data Availability

The original contributions presented in the study are included in the article/Supplementary material, further inquiries can be directed to the corresponding author.
