# Supplementary material for: Within-subject and between-subject variability in responses to a mind-body intervention in electrophysiological brain activity
Source: Front Psychol. 2026 Jul 22;17:1872803. doi: 10.3389/fpsyg.2026.1872803 (PMC13437499; doi:10.3389/fpsyg.2026.1872803)
Supplement: Supplementary file 1 [file Supplementary_file_1.docx]

(Introduction:) Close your eyes now - still sitting. There are a few things you should know before we begin.

When you go somewhere to do something good for yourself, there is usually someone there to tell you what is right and what is wrong. And if you don't do it the way it is supposed to be done, they will correct you. Here it's different. You have to find out your own way how to do it best. Everyone begins their learning process at their own starting point. That's why it's not possible that the same is right for everyone. So just do what is easy and comfortable for you, regardless of what I say or suggest. This is especially true if anything I suggest causes pain or increases existing pain. Then just don't do it. Pain is a warning signal. Your body shows you the limits within which you can move safely. Under no circumstances should you exceed them.

The thought alone, the idea of what you want to do, is enough to initiate the actual motion. This first impulse, the inner image of what you want to do, already contains all the elements you need to complete the movement. Simply visualise what you want to do and the process begins. We will take a short break every few minutes for about 1 minute. These breaks are as important as what we do between breaks. They give your brain the rest it needs to compare what you have been doing with your previous experiences, integrate new things and confirm what you already know.

Posture and movement are controlled unconsciously. That's why everything we do is directed to the unconscious. Whether you perceive something consciously or not, you can be sure: Your unconscious perceives every change, no matter how small. To further develop your own skills, the most important thing is to know where you are starting your journey. Only then can you look for paths that lead you in the direction you want to go. There is a very simple way to do this. The first step: Observe how you do what you do. The second step: play with different variations, try out how else you could do what you do, how else. And in the third step: return to the original movement and observe how you now do what you do. In this way, you can improve the quality of your actions step by step. And that doesn't just apply to movement.

(Break 0:) Now lay down on your back. Time to feel. Time to make a picture of your situation. A white sheet of paper on which your body is laying leaves an imprint in the color or colors of your choice. Where it rests firmly, the imprint is stronger, where it rests less firmly, it is weaker. And then there are areas where your leaf remains white. Where can you feel changes anywhere that are related to your respiration - in and out? Changes in the contact between your body and the floor, changes in the relationship between the parts of your body - sternum, ribs, spine - and changes in the relationship to space.

(Task 1:) If you now put both legs, arms and hands on the floor, to the left and right side of your body, you can now begin to gently press one elbow and one foot against the floor at the same time in one direction of breathing, release the pressure when your breath reverses, and repeat the movement in the rhythm of your respiration: press a little and release again. And observe which shoulder and which hip starts to lift a little bit when foot and elbow press against the floor. Lift a little bit when the elbow and foot press, and the hip and shoulder lower again when foot and elbow release the pressure. The one when you breathe in one direction, the other when the breath turns back. And now switch and move the other foot and the other elbow. The two now press against the floor at the same time and release the pressure again. And observe which shoulder and which hip now want to participate and allow to be lifted a little bit. Now move on to alternate directly between one elbow and foot, then the foot and elbow of the other side, alternating. Observe how this movement now affects your hips and shoulders. Now leave the actual movement, and you can just imagine - only imagine - these movements. Feet, elbows, shoulders and hips, only in your imagination. Feet, elbows, shoulders and hips only in your imagination. If you now switch back from the imagination to the actual movement again: one foot, one elbow, press a little against the floor, at the same time, one shoulder and one hip lift a little, return when the elbow and foot release the pressure, and so on, in this calm rhythm of breathing in and breathing out. When you now switch back to the other elbow and the other foot, the same movement, foot and elbow press simultaneously, while one shoulder and one hip lift a little bit at the same time. Now switch directly to foot and elbow of the other side, then the other, and the other. And see what happens to your hips, pelvis and shoulders. Push and release, lift and return.

(Break 1:) Now put both legs down and take some time to rest. And resting can always also mean: experiencing how you are lying now. Time to rest and time to explore. Time for your unconscious to compare the new with the familiar. Time to rest and time to feel. These little breaks that our nervous system needs.

(Task 2:) Now put up your legs again. You can put your hands on your belly and imagine a small clock face above your nose and eyes. And while your head remains still, your eyes can start to move. In one direction to one side and back to the starting point and to the other side and back again, so that the eyes now roll from the starting point to the 3, back to the starting point and then back to the 9 and back to the starting point. Always take a full breath from the starting point to the turning point and back. The nose remains focused on the starting point. The head lies calmly there. What impulses does your head receive? Without picking them up. If you now go back to visualizing a point at the ceiling, with your eyes closed, your head can start to roll. The nose to one side with one direction of breathing, back to the starting point when the breath reverses. With the next breath, it moves to the other turning point and returns again. A whole breath time between the starting point and the three, between the starting point and the nine. Your head rolls and your eyes stay in place so that your head can now roll behind your eyes. Instead of the eyes in relation to the head as before, now let eyes and head both roll to one side and back, then to the other side and back again. Breathe in and out as your eyes and nose move to one side and then back to the other. When breathing in one direction, they move away from the starting point. When the breath reverses, they return. Breathe in and out. Move your eyes and head to one side and back, then to the other side and back again. To one side and back, to the other side and back. A whole breath of time.

(Break 2:) Now put your legs down, you can rest again, take time to rest, take your time. Give yourself time to breathe in and out calmly. These little breaks. Rest a little, again and again. Feel how you are lying now. And resting can always also mean: exploring how you are lying now. In this calm rhythm of your breath.

(Task 3:) Once you have put your legs up again, you can place your hands on the lower ribs from the left and right, the left one on the left, the right one on the right, and observe how the ribs lift the hands when you breathe in and allow them to return when you breathe out. And more and more you can direct your breath to where your hands are, so that they are lifted when you breathe in and can sink back when you breathe out. Your head and eyes can begin to roll. To one side with one direction of breath, back when the breath turns back. With the next breath to the other side and back again, so that your head and eyes roll once to the 3 and back, once to the 9 and back in this calm rhythm of your respiration. And if you now, while your eyes and head continue to roll, place your hands on your upper ribs on the left and right side, the left hand on the left side, the right hand on the right side, then you can redirect your breath to where your hands are now. They are lifted by the ribs when you breathe in and sink with the ribs when you breathe out. And so, in this calm rhythm of your breathing. Now leave the movement for two breaths, cross your arms so that your left hand is on the lower ribs on the right side and your right hand is on the left side. And let your hands intensify the contact with your ribs a little. Press against them a little. And observe how breathing in and out move the ribs, how the hands are lifted a little when you breathe in and sink back together with the ribs when you breathe out. And then your head can start to move to one side when you breathe in one direction, back when the breath reverses, and then to the other side and back. Between the starting point and the 9, back, and then to the 3 on the clock face, and back again. If you leave this movement again for a breath or two, you can now cross your arms the other way round and place them high up on your chest. The left hand on the right and the right hand on the left. Your breath expands your chest and allows it to become smaller again, to sink. Lift your hands and lower them together with your ribs. The eyes and your head roll to one side, 3 or 9, and back when the breath reverses and then to the other side and back again. A whole breath of time, from the starting point to the turning point and back again. Hands and ribs, breathing in and breathing out. What is different, now that your hands are up there instead of on your lower ribs.

(Break 3:) Now, put everything down again, give yourself time to reach the ground. Time to rest. And while one part is busy resting, another part can observe how you are lying now. Breathe in and out. Rest and feel.

(Task 4:) If you now put your legs up again, imagine a diagonal line between your left shoulder and your right hip. And when you imagine this line, what do your eyes do? If you then look at the other diagonal line, between your right shoulder and left hip, and your eyes. Where do the two diagonals cross? And where have you placed them? On the front or back of your body? Are they lying on you or are you lying on them? When you observe your inhalation and exhalation, you can now - with your eyes still closed - begin to move one eye forwards towards the point on the ceiling where your gaze is focused, while the other eye simultaneously withdraws into its cave. Both reverse when the breath reverses, and then vice versa. The other eye moves forwards while the other withdraws. And so on in direct alternation. With one breath, one eye moves forwards, the other withdraws. On the next breath, vice versa. At the same time, one eye in one direction, the other in the other. Opposite. One forwards, the other backwards. And how do you deal with it when something doesn't go quite as easily as you would like it to? You can do the same eyes forwards a few times when the other one pulls back. A few times the same eyes forwards, the other backwards. A few times, and then the other way round: the eye that was moving forwards now pulls back when the other one moves forwards. Move away from the starting point in one breathing direction and return when the breath reverses.

(Break 4:) If you now put your legs down in the way that is most comfortable for you, you can visualise your position once more, once more: time to feel. A white sheet on which your body leaves an imprint in the color or colors of your choice. Where it rests firmly, the imprint is stronger, where it rests less firmly, it is weaker. And then there are the areas where your leaf remains white. Where can you feel changes everywhere that are related to your breath - in and out? Changes in the contact between your body and the ground, changes in the relationship between the parts and changes in the relationship to the space.

(Exit:) And when you are ready, you can open your eyes, sit up and arrive back in the here and now.
